# Hepatoprotective Potential of *Clitoria ternatea* Leaf Extract Against Paracetamol Induced Damage in Mice

**DOI:** 10.3390/molecules161210134

**Published:** 2011-12-06

**Authors:** Kuppan Nithianantham, Murugesan Shyamala, Yeng Chen, Lachimanan Yoga Latha, Subramanion L. Jothy, Sreenivasan Sasidharan

**Affiliations:** 1 Department of Biotechnology, Faculty of Applied Sciences, AIMST University, Jalan Bedong-Semeling, Batu 3½, Bukit Air Nasi, Bedong, 08100 Kedah, Malaysia; 2 Dental Research & Training Unit, and Oral Cancer Research and Coordinating Centre (OCRCC), Faculty of Dentistry, University of Malaya, 50603 Kuala Lumpur, Malaysia; 3 Institute for Research in Molecular Medicine (INFORMM), Universiti Sains Malaysia, 11800 Pulau Pinang, Malaysia; Email: latha_usm@yahoo.com (L.Y.L.); srisasidharan@yahoo.com (S.S.)

**Keywords:** *Clitoria ternatea*, paracetamol, hepatoprotective, antioxidant activity

## Abstract

Background and Aim: *Clitoria ternatea*, a medicinal herb native to tropical equatorial Asia, is commonly used in folk medicine to treat various diseases. The aim of the present study is to evaluate the hepatoprotective and antioxidant activity of *C. ternatea* against experimentally induced liver injury. Methods: The antioxidant property of methanolic extract (ME) of *C. ternatea* leaf was investigated by employing an established *in vitro* antioxidant assay. The hepatoprotective effect against paracetamol-induced liver toxicity in mice of ME of *C. ternatea* leaf was also studied. Activity was measured by monitoring the levels of aspartate aminotransferase (AST), alanine aminotransferase (ALT) and billirubin along with histopathological analysis. Results: The amount of total phenolics and flavonoids were estimated to be 358.99 ± 6.21 mg/g gallic acid equivalent and 123.75 ± 2.84 mg/g catechin equivalent, respectively. The antioxidant activity of *C. ternatea* leaf extract was 67.85% at a concentration of 1 mg/mL and was also concentration dependant, with an IC_50_ value of 420.00 µg/mL. The results of the paracetamol-induced liver toxicity experiments showed that mice treated with the ME of *C. ternatea* leaf (200 mg/kg) showed a significant decrease in ALT, AST, and bilirubin levels, which were all elevated in the paracetamol group (*p* < 0.01). *C. ternatea* leaf extract therapy also protective effects against histopathological alterations. Histological studies supported the biochemical findings and a maximum improvement in the histoarchitecture was seen. Conclusions: The current study confirmed the hepatoprotective effect of *C. ternatea* leaf extract against the model hepatotoxicant paracetamol. The hepatoprotective action is likely related to its potent antioxidative activity.

## 1. Introduction

*Clitoria ternatea* L. (CT) (Family: Fabaceae) is an ornamental perennial climber, up to 2–3 m in height, bearing conspicuous blue or white flowers resembling a conch shell and found growing in the wild and also in cultivated gardens [1]. The leaves and roots are used in the treatment of a number of ailments including body aches, infections, urinogenital disorders, and as an anthelmentic and antidote to animal stings [2]. The young shoots, leaves, flowers and tender pods are eaten as a vegetable in Kerala (India) and in the Philippines. In Malaysia, the leaves are employed to impart a green color to food and the flowers to impart a bright blue color to rice cakes [1]. Furthermore, the leaves are useful in otalgia and hepatopathy, whereas seeds are cathartic Anonymous, 1995 Anonymous, *Indian medicinal plants*, vol. 2 Orient Longman, Madras (1995), pp. 129–132. [3]. Moreover, the root, stem and flower are recommended for the treatment of snakebites and scorpion stings in India [4]. Various pharmacological activities of *C. ternatea* were reported in literature such as antimicrobial, antipyretic, anti-inflammatory, analgesic, diuretic, local anaesthetic, antidiabetic, insecticidal, blood platelet aggregation inhibiting and vascular smooth muscle relaxant properties [1]. *C. ternatea* is commonly used in Ayurvedic medicine to treat various types of ailments including as memory enhancer, nootropic, antistress, anxiolytic, antidepressant, anticonvulsant, tranquilizing and sedative agent [1]. Various secondary metabolites such as polyphenolic flavonoids, anthocyanin glycosides, pentacyclic triterpenoids and phytosterols have been reported from this plant. Flavonols *i.e.*, kaempherols, quercetin and myricetin and their glycosides were also isolated from this plant [5]. Since various antioxidant compounds were isolated from this plant, *C. ternatea* has good potential for development as an antioxidant and hepatoprotective agent.

Despite remarkable advances in modern medicine, hepatic disease remains a worldwide health problem, thus the search for new medicines is still ongoing. Hepatic cells participate in a variety of metabolic activities, therefore the development of liver protective agents is of paramount importance in the protection from liver damage. The literature has constantly shown that hepatoprotective effects are associated with plant extracts rich in antioxidants [6,7,8,9,10]. Many compounds and extracts from plants have thus been evaluated for hepatoprotective and antioxidant effects against chemically-induced liver damage [11,12,13]. Moreover, research on hepatoprotective medicinal plants as a major indicator of the general screening systems can trigger the safety evaluation in the early phase of drug discovery because most of the toxic compounds are metabolized in liver [14,15,16,17,18]. To the best of our knowledge, the hepatoprotective effect of *C. ternatea* leaves against paracetamol-induced liver injury in mice has not been demonstrated. Hence, the present study focused on evaluating the potential hepatoprotective effect of methanolic extract from *C. ternatea* leaves on paracetamol-induced liver injury in mice.

## 2. Results and Discussion

### 2.1. Radial Scavenging (DPPH) Assay and IC_50_ Determination

**Figure 1:**
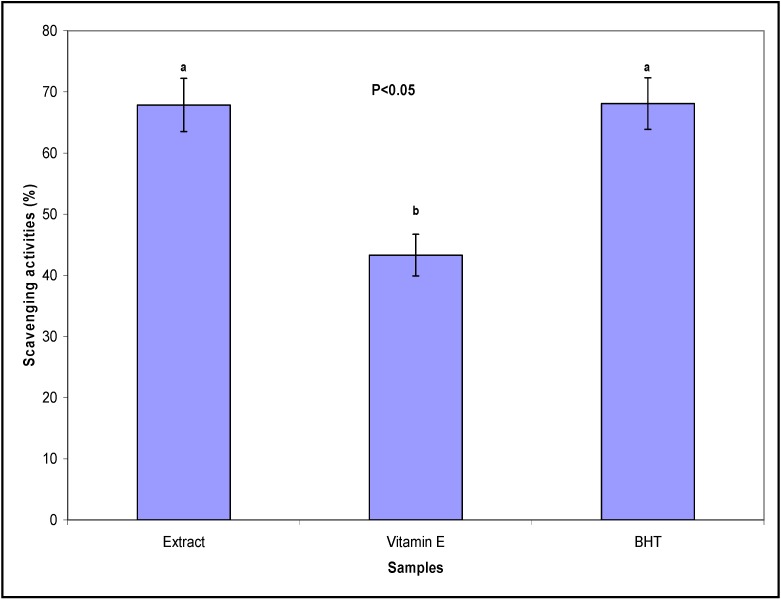
Scavenging effect (%) of extract of *Clitoria ternatea*, and standard antioxidants, butylated hydroxytoluene (BHT) and vitamin E at 1.0 mg/mL.

The antioxidant potential of methanolic *C. ternatea* leave extract was investigated in this with the aim of identifying potentially new antioxidant bioactive medicinal plant extracts. The measured DPPH radical scavenging activity is summarized in Figure 1. The percentages of free radical scavenging activities were 67.85%, 43.30% and 68.10% for the *C. ternatea* extract, vitamin E and BHT, respectively. The IC_50_ value, which is the concentration of crude extract that decreases the initial DPPH radical concentration by 50%, was 420.00 µg/mL. As mentioned above, flavonols their glycosides have been isolated from *C. ternatea* [5], and these flavonols may be responsible for the good antioxidant activity observed in this study. 

### 2.2. Total Phenolic and Flavonoid Content

Phenolic and flavonoid compounds have been reported to be responsible for the antioxidant activity observed in many plant extracts [19]. The total phenolic content of *C. ternatea* extract was determined to be 358.99 ± 6.21 mg/g gallic acid equivalent. In addition, the total flavonoid content of *C. ternatea* extract was 123.75 ± 2.84 mg/g catechin equivalent. Thus the free radical scavenging activity exhibited by *C. ternatea* extract could be attributable to the presence of these phenolic and flavonoid compounds in the extract.

### 2.3. Biochemical Parameters

The effects of *C. ternatea* extract on liver marker enzymes and serum bilirubin content are given in Table 1. The data showed that the control group demonstrated a normal range of AST, ALT and billirubin levels, while the paracetamol-treated group showed elevated levels of AST, ALT and bilirubin, confirming that paracetamol caused liver injury at higher doses. The elevation of cytoplasmic AST and ALT is considered an indicator for the release of enzymes from disrupted cells. Bilirubin concentration has been used to evaluate chemically induced hepatic injury. Besides its various normal functions, the liver excretes the breakdown product of hemoglobin, namely bilirubin, into bile. It is well known that necrotizing agents like paracetamol produce sufficient injury to the hepatic parenchyma to cause large increases in bilirubin content [20]. On the other hand, the extract-treated group showed a very interesting result. Based on the Table 1 data, the biochemical parameters of the extract treated group were higher than those of the control group (*p* < 0.05), but it showed much lower levels of AST, ALT and billirubin than the paracetamol-treated group, that is, the extract treatment significantly reduced the previously raised levels of AST, ALT and billirubin in hepatotoxic mice. The decrease in the serum levels of these enzymes might be due to the presence of various phenolic and flavonoid compounds in the leaf extract that enhanced the liver’s regeneration ability. One way Analysis of Variance (ANOVA) was done, where p is less than 0.05 and n = 6 to prove the significance of the obtained results.

**Table 1:** Effect of *C. ternatea* extract on liver marker enzymes and serum bilirubin content.

Parameters	Control	Paracetamol Treated	Extract Treated
AST (IU/L)	38.83 ± 5.32	108.65 ± 12.21 **	42.1 ± 6.25 *
ALT (IU/L)	32.58 ± 6.12	98.23 ± 10.12 **	39.23 ± 5.32 *
Billirubin (mg/L)	1.5 ± 0.5	8.7 ± 3.9 **	2.3 ± 1.3 *

Results are expressed as mean ± S.E.M; * Statistically significant compared to paracetamol treated animals (*p* < 0.05); ** Statistically significant to control animals (*p* < 0.05).

### 2.4. Histopathology Analysis

**Figure 2:**
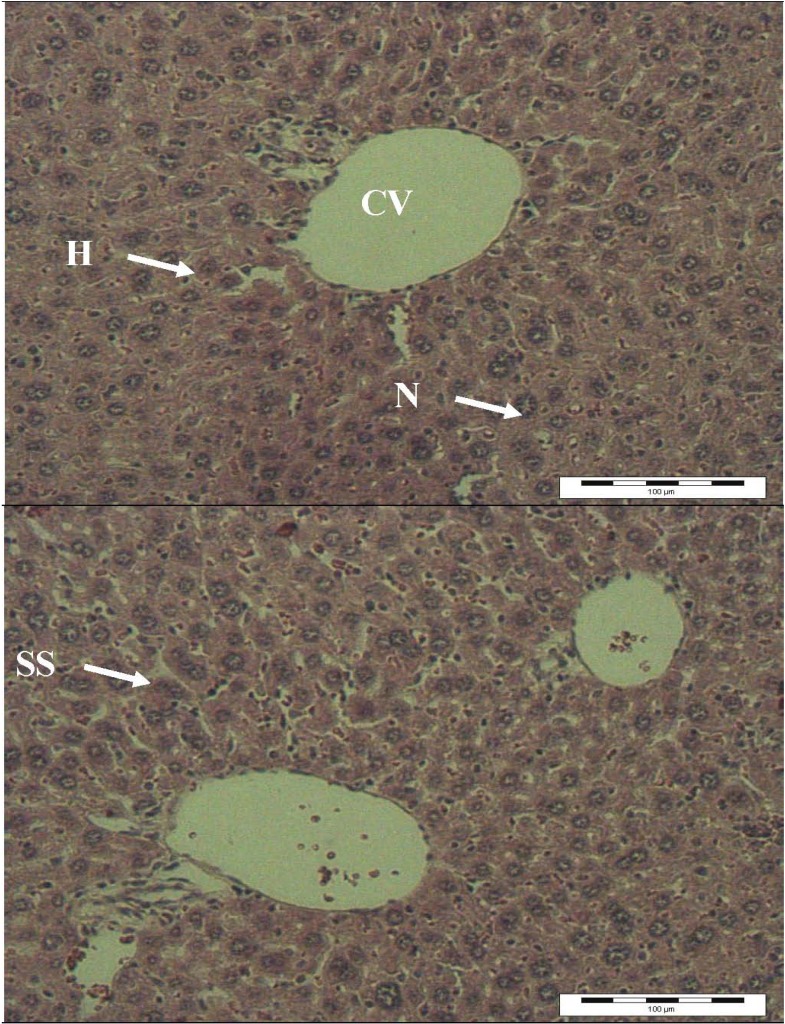
Light microphotographs of liver cell of normal mice. (H: hepatocytes; N: nucleus; SS: sinusoid; CV: central vein).

**Figure 3:**
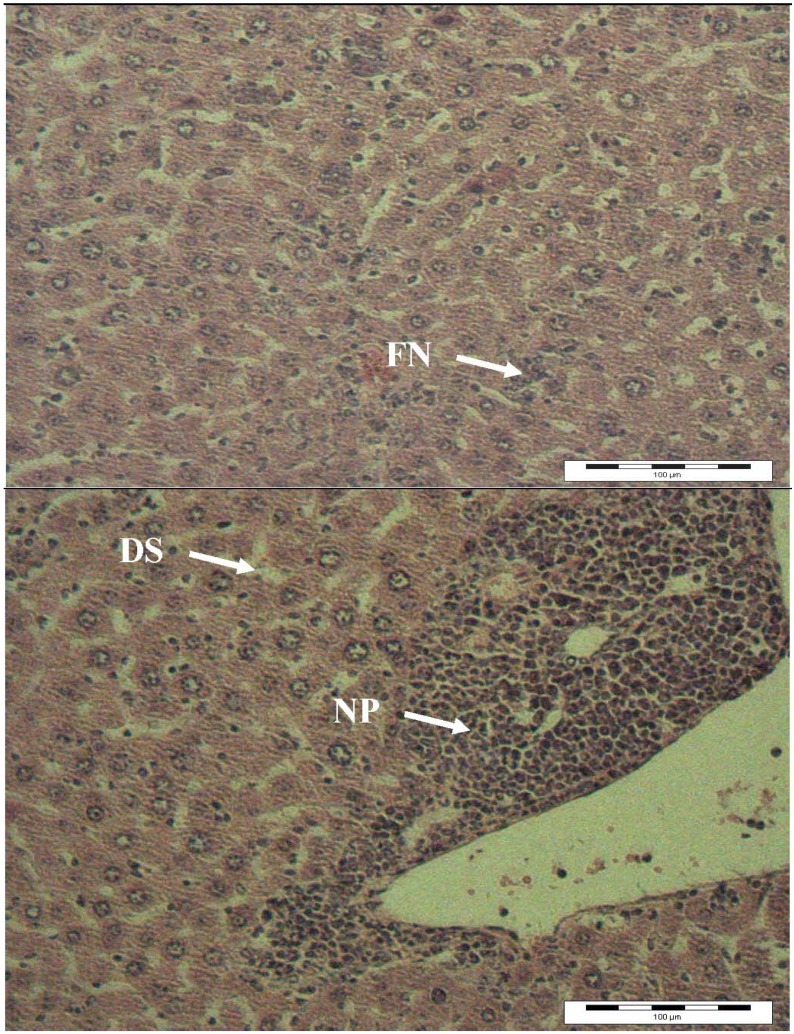
Light microphotographs of liver cell of mice exposed to paracetamol. (NP: Neutrophil; DS: dilated sinusoid; FN: focal necrosis).

**Figure 4:**
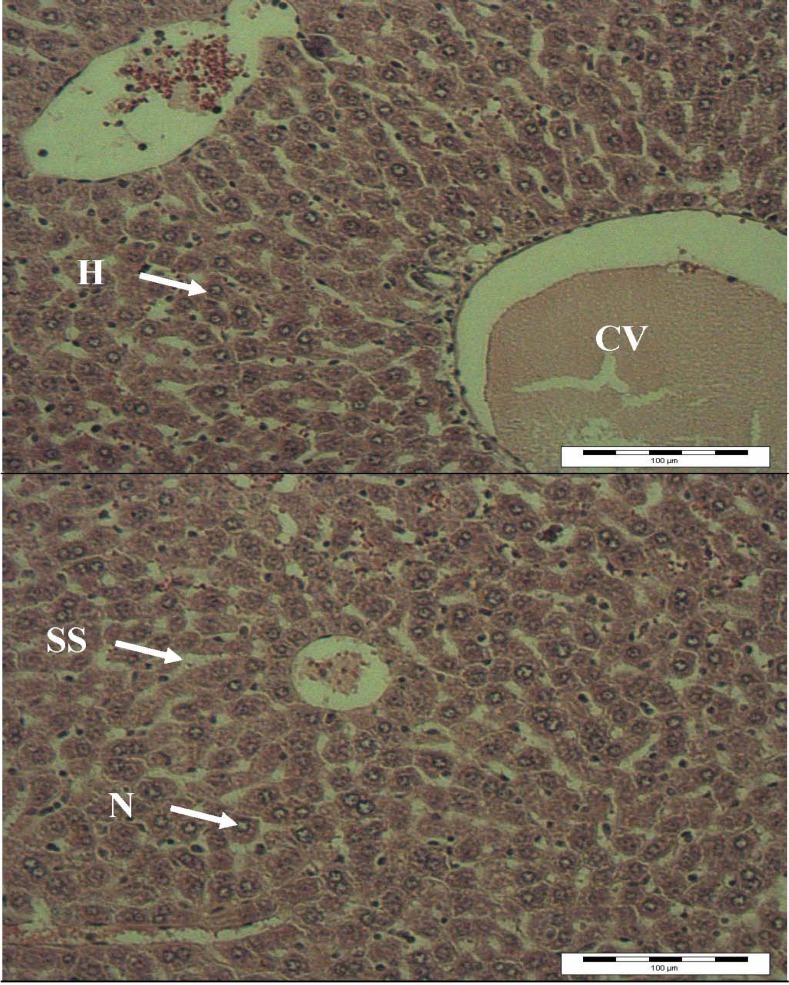
Light microphotographs of liver cells of mice treated with *C. ternatea* extract. (H: hepatocytes; N: nucleus; SS: sinusoid; CV: central vein).

The results of light microscopy examination of the transverse section of control, paracetamol-treated and extract-treated mice livers are shown in Figure 2,Figure 3,Figure 4. Figure 2 shows the liver cells of mice in the control group. From that image it can be observed that the liver cells are normal in shape, the cell nuclei are intact and most importantly, the portal vein has a regular shape. Overall, a healthy set of cells can be observed. Figure 3 shows the liver of paracetamol intoxicated mice showing wide necrosis across the cells. The liver sections of these mice showed necrosis, ballooning and degeneration in hepatic plates and loss of cellular boundaries. There was also a heavy accumulation of neutrophils surrounding the portal vein. These neutrophils act as an indicator of the occurrence of cell damage as they are absent in normal healthy tissues. The hepatocytes are disrupted and sinusoids are damaged as well. Figure 4 shows the histological architecture of treated liver sections with a mild degree of degeneration and necrosis. The hepatocytes nucleases are at a recovery stage and there are very minimal numbers of neutrophils surrounding the portal vein. Therapy with *C. ternatea* extract was effective in restoring the paracetamol induced histopathological lesions and almost normal liver architecture was exhibited, with well-formed hepatocytes separated by sinusoids and maintained cord arrangement. The histopathological examination thus verified the hepatoprotective effect of *C. ternatea* extract against the model hepatotoxicant paracetamol.

## 3. Experimental

### 3.1. Sample Collection

Fresh leaves of *C. ternatea* were collected from various areas in AIMST University, Kedah, Malaysia in January 2011. The leaves were separated and cut into small pieces, which were first washed with tap water and then with distilled water. The leaves were then dried in an oven at 60 °C for 7 days, after which the dried leaves were ground into fine powder using a grinder.

### 3.2. Extraction Procedure

Dried sample (approximately 100 g) was added to methanol (300 mL) and soaked for 4 days at room temperature (30 ± 2 °C). The suspension was stirred from time to time to allow the leaf powder to fully dissolve in the methanol. Removal of the sample from the solvents was done by filtration through cheesecloth followed by filter paper (Whatman No. 1); the filtrate was concentrated under vacuum to one-fifth its volume using a rotary evaporator at 60 °C and then sterilized by filtration using a 0.22-mm membrane. The thick paste obtained was further dried in an oven at 40 °C. The resultant extract was kept at 4 °C for further analysis. The methanol was used for the extraction in this study to mimic the usage of water by the traditional healers to prepare plant extract as a decoction. Water and methanol have the highest polarity in the polar protic solvent group. Moreover, the usage of methanol makes the process of evaporation easier compared to water.

### 3.3. Antioxidant Activity Assays

2,2-Diphenyl-1-picrylhydrazyl radical (DPPH) scavenging assay quantitative measurement of radical scavenging activity (RSA) was carried out in a universal bottle. The reaction mixture contained test sample (50 μL) ranging in concentration from 0.5 mg/mL to 6 mg/mL and 0.004 w/v % DPPH solution in methanol (5 mL, 80% v/v). The mixture without test sample was used as blank and spiked with blank methanol (50 μL). The commercial antioxidants butylated hydroxytoluene (BHT, Sigma) and vitamin E were used for comparison or as a positive control. Discoloration was measured at 517 nm after incubation for 30 min. Measurements were taken in triplicate. The DPPH radical concentration was calculated using the following equation: DPPH scavenging effect (%) = A_0_ − A_1_/A_0_ × 100 where A_0_ is control absorbance and A_1_ is the absorbance in the presence of the sample (mushroom extract) [21]. The actual decrease in the absorbance induced by the test sample was compared with the positive controls.

### 3.4. IC_50_ Determination

The IC_50_ was defined as the amount of the sample that is sufficient to elicit 50% reduction of the initial DPPH radical concentration. This was calculated from the linear part of the inhibition of DPPH radical [22].

### 3.5. Determinations of Total Phenolic Contents

Total phenolic content of *C. ternatea* leaf extract was determined using the Folin-Ciocalteau assay according to the previously described method [23]. Total phenolic content was calculated from the calibration curve of a gallic acid standard solution. Results were expressed as gallic acid equivalents, in mg/g dry extract.

### 3.6. Determination of Total Flavonoid Content

The amount of total flavonoid in the *C. ternatea* leaf extract was measured spectrophotometrically following method described by Djeridane *et al.* [24]. Catechin was used as standard to construct a calibration curve. Various concentrations of *C. ternatea* leaf extract (1.5 mL) were mixed with of 2% methanolic AlCl_3_ solution (1.5 mL). After incubation at room temperature for 15 min, the absorbance of the reaction mixture was measured at 430 nm. The flavonoids content was expressed as mg catechin equivalents (CE) in 1 g extract (mg CE/g).

### 3.7. Hepatoprotective Activity of C. ternatea Leaf Extract

#### 3.7.1. Animals

Eighteen specific pathogen free and age-matched (7- to 10-week-old) male Wister albino mice were used to study the hepatoprotective activity of the *C. ternatea* leaf extract. The Institution Animal Ethics Committee of AIMST University has approved the animal study for this project. The animals were kept at 27 ± 2 °C, relative humidity 44–56% and light and dark cycles of 10 and 14 h respectively, for a week before and during the experiments. Animals were provided with standard diet (Lipton, India) and water *ad libitum*. The food was withdrawn 18–24 h before starting the experiment. All experiments were performed in the morning according to current guidelines for the care of the laboratory animals and the ethical guidelines for the investigation of experimental pain in conscious animals [25].

#### 3.7.2. Paracetamol Dose Regimen

Paracetamol tablets were obtained from a nearby clinic. Each tablet contains 500 mg of paracetamol. The dose administered to the mice to induce hepatotoxicity was set as 1 g/kg. The paracetamol was turned into a fine powder using a mortar and pestle to increase the dissolution. The powdered paracetamol was suspended in saline and was administered orally according to the body weight of mice.

#### 3.7.3. Grouping of Mice and Treatments

Eighteen mice (25–30 g) were randomly divided into three groups, each group consisting of six mice. The first group received a single daily dose of 1 mL/kg of saline orally (control group). Group II was given a single daily dose of paracetamol (1.0 g/kg) orally (induced group) and Group III received orally a single daily dose of both 1.0 g/kg paracetamol [26] and 200 mg/kg of *C. ternatea* leaf extract respectively (treated group). *C. ternatea* leaf extract was administered three hours after the administration of paracetamol. The treatments were continued for seven days and on the eighth day of the experiment all animals were anesthetized and dissected [27].

#### 3.7.4. Sacrifice and Organ Harvesting

The liver was removed carefully after euthanizing and killing the animals by cervical dislocation. The livers were fixed in 10% buffered formalin. After fixation, the livers were dehydrated in a graded series of alcohol, cleared in xylene and embedded in paraffin wax. Multiple 5 μm sections from each block were mounted on slides and stained with hematoxylin and eosin.

#### 3.7.5. Biochemical Parameters

The mice of each group were anaesthetized with ether, and blood was collected directly from the heart. It was centrifuged at 2,000 g for 10 min at 4 °C to separate the serum and kept at 4 °C to assay the activities of serum enzymes. Aspartate aminotransferase (AST) and alanine aminotransferase (ALT) were determined by the method described by Reitman and Frankel, [28]. Serum bilirubin level was estimated according to Malloy and Evelyn [29].

#### 3.7.6. Statistical Analysis

All values are mean ± S.E.M. obtained from six animals. For statistical analysis, one-way ANOVA with Duncan’s variance (SPSS 15) was used to compare the groups. In all the cases a difference was considered significant when *p* < 0.05.

## 4. Conclusions

In the present study, methanolic *C. ternatea* leaf extract possessed strong hepatoprotective and antioxidant activity in a mouse model of paracetamol-induced hepatotoxicity. The hepatoprotective activity of *C. ternatea* leaf may be due to its free radical-scavenging and antioxidant activity, resulting from the presence of some phenolic compounds in the extracts. Further studies are in progress to better understand the mechanism of action of *C. ternate* responsible for the observed hepatoprotective and antioxidant activity.
